# Associations of longitudinal D-Dimer and Factor II on early trauma survival risk

**DOI:** 10.1186/s12859-021-04065-z

**Published:** 2021-03-13

**Authors:** Richard M. Jiang, Arya A. Pourzanjani, Mitchell J. Cohen, Linda Petzold

**Affiliations:** 1 Department of Computer Science, University of California, Santa Barbara, Santa Barbara, USA; 2 Now at Foresite Capital, San Francisco, USA; 3 Denver Hospital, Denver, USA

**Keywords:** Early trauma survival risk, Longitudinal models, Joint models, Clinical panel data, D-Dimer, Factor II

## Abstract

**Background:**

Trauma-induced coagulopathy (TIC) is a disorder that occurs in one-third of severely injured trauma patients, manifesting as increased bleeding and a 4X risk of mortality. Understanding the mechanisms driving TIC, clinical risk factors are essential to mitigating this coagulopathic bleeding and is therefore essential for saving lives. In this retrospective, single hospital study of 891 trauma patients, we investigate and quantify how two prominently described phenotypes of TIC, consumptive coagulopathy and hyperfibrinolysis, affect survival odds in the first 25 h, when deaths from TIC are most prevalent.

**Methods:**

We employ a joint survival model to estimate the longitudinal trajectories of the protein Factor II (% activity) and the log of the protein fragment D-Dimer ($\upmu$g/ml), representative biomarkers of consumptive coagulopathy and hyperfibrinolysis respectively, and tie them together with patient outcomes. Joint models have recently gained popularity in medical studies due to the necessity to simultaneously track continuously measured biomarkers as a disease evolves, as well as to associate them with patient outcomes. In this work, we estimate and analyze our joint model using Bayesian methods to obtain uncertainties and distributions over associations and trajectories.

**Results:**

We find that a unit increase in log D-Dimer increases the risk of mortality by 2.22 [1.57, 3.28] fold while a unit increase in Factor II only marginally decreases the risk of mortality by 0.94 [0.91,0.96] fold. This suggests that, while managing consumptive coagulopathy and hyperfibrinolysis both seem to affect survival odds, the effect of hyperfibrinolysis is much greater and more sensitive. Furthermore, we find that the longitudinal trajectories, controlling for many fixed covariates, trend differently for different patients. Thus, a more personalized approach is necessary when considering treatment and risk prediction under these phenotypes.

**Conclusion:**

This study reinforces the finding that hyperfibrinolysis is linked with poor patient outcomes regardless of factor consumption levels. Furthermore, it quantifies the degree to which measured D-Dimer levels correlate with increased risk. The single hospital, retrospective nature can be understood to specify the results to this particular hospital’s patients and protocol in treating trauma patients. Expanding to a multi-hospital setting would result in better estimates about the underlying nature of consumptive coagulopathy and hyperfibrinolysis with survival, regardless of protocol. Individual trajectories obtained with these estimates can be used to provide personalized dynamic risk prediction when making decisions regarding management of blood factors.

## Background

Coagulopathy (as defined here) is a condition in which blood fails to properly form robust clot. Following injury and shock from a major trauma, patients become coagulopathic, coinciding with increased bleeding, higher resuscitation requirements and much higher rates of death [1–3]. However, despite the increased urgency for treatment, the complexity of the underlying coagulation system makes understanding and diagnosis of trauma-induced coagulopathy (TIC) extremely difficult, especially in a clinical setting with so much interpatient and intrapatient variability. The main objective of this study is to quantify the level to which markers of two possible mechanisms of TIC affect survival odds, accounting for patient variability, and to understand what this tells us about possible targets for intervention.

### The coagulation system and coagulopathy

**Fig. 1:**
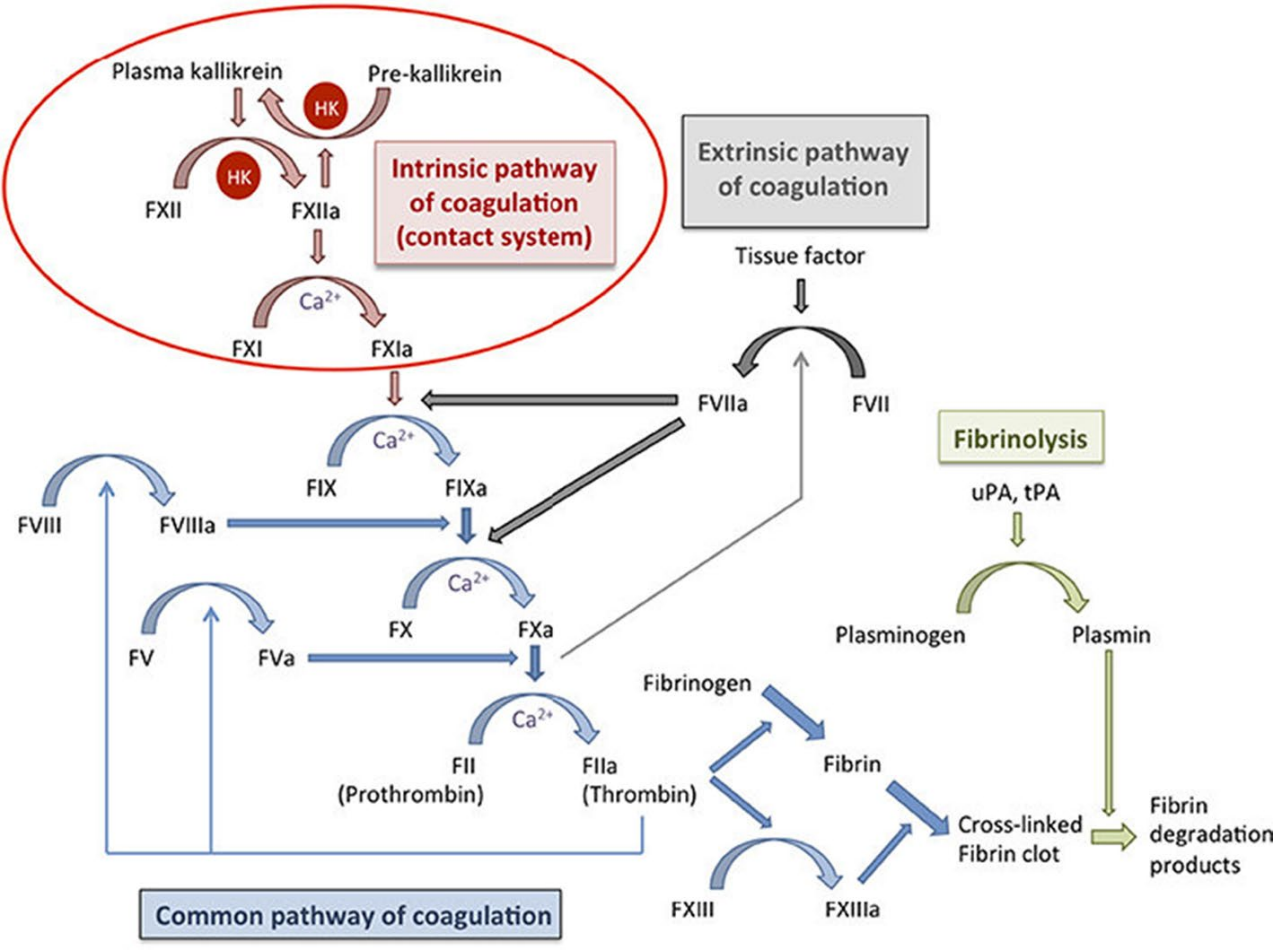
The coagulation cascade and fibrinolysis [5]. The coagulation cascade is responsible for formation of a fibrin clot, while fibrinolysis is resposible for breaking down fibrin clots. Balance in the system is crucial for the regulation of overall coagulation

The standard model for the coagulation system consists of two distinct physical processes: coagulation (clot formation) and fibrinolysis (clot breakdown). Coagulation is the process by which a sequence of protein interactions ultimately leads to the formation of cross-linked fibrin clots, which physically block off a wound site [4]. To balance this process, fibrinolysis breaks down fibrin clots and produces fibrin degradation products, which are then flushed out of the system. Properly regulated, these two systems prevent excessive bleeding. A schematic is shown in Fig. 1.

Malfunctions in the coagulation system lead to the inability to form clots or to keep clots in place, resulting in excessive bleeding at the wound site. Several hypotheses exist to explain the driving factors of TIC [6, 7]. Two important coagolopathic conditions are consumptive coagulopathy and hyperfibrinolysis. Consumptive coagulopathy focuses on the inability to form fibrin clots, due to a lack of necessary pro-coagulants, while hyperfibrinolysis emphasizes the inability to keep a sufficient number of fibrin clots active due to overactive fibrinolysis. Though the mechanisms are different, both manifest as increased, uncontrollable bleeding at the wound, often through a complex interdependent mechanism.

In this study we used data collected from trauma patients to quantify how these two mechanisms may be realized in patient survival odds. We chose Factor II and D-Dimer as representative biomarkers of consumptive coagulopathy and hyperfibrinolysis respectively. Factor II, or prothrombin, is a protein that is converted into thrombin in the coagulation cascade [8]. Thrombin is the central protein in the coagulation cascade, responsible for forming fibrin clots and activating platelets to essentially seal a wound. On the other hand, D-Dimer is a fibrin degradation product created when plasmin breaks down fibrin clots. We fit a joint survival model to this data and examined the distribution of patient longitudinal curves and the hazards of both longitudinal covariates.

## Methods

### Dataset

Our dataset consists of severely injured patients admitted to the ICU at the UCSF Level I Trauma Center. Upon admission, age, sex, injury severity score, injury type, and the presence of a traumatic brain injury, in addition to many other measurements, were recorded. Blood draws were attempted for each patient at hours close to 0, 2, 3, 4, 6, 12, and 24 as measured from admission. The time and outcome of each patient was recorded post dispatch. From each blood draw, a variety of coagulation activity levels were measured, of which only the protein Factor II (% activity) and the protein fragment D-Dimer ($\upmu$g/ml) were used in this analysis, for the aformentioned reasons. Blood assays were conducted using the Stago Compact Analyzer (Diagnostica Stago, Parsippany, NJ) according to manufacturer instructions. For D-Dimer, the upper limit normal value is $\sim$0.5 $\upmu$g/ml [9] while for Factor II standard operating range falls within 50–200% activity. The hour 0 measurements of most patients fell within these values though with a slight skew due to the nature of the dataset. Patients with no Factor II or D-Dimer measurements were omitted. Post pre-processing, a total of 891 patients remained with 2062 longitudinal observations. In this work we define outcome as survival at hour 25, which is on the order of when deaths from TIC are most prevalent [10]. Past this window, many patients die from other causes such as sepsis. From a survival analysis perspective, patients were considered censored if death was not recorded within the observation window. A summary of the distributions in the data are presented in Table 1.

**Table 1:** Characteristics of cohort

Characteristic	Estimate
Total number of individuals	891
Death within 24 h, n (%)	61 (6.8%)
Sex, n (%)	
Male	728 (81.7%)
Age, n (%)	
$\ge$ 15, < 20	58 (6.5%)
$\ge$ 20, < 30	286 (32.1%)
$\ge$ 30, < 40	170 (19.1%)
$\ge$ 40, < 50	127 (14.3%)
$\ge$ 50, < 60	115 (12.9%)
$\ge$ 60, < 70	62 (6.9%)
$\ge$ 70, < 80	42 (4.7%)
$\ge$ 80	31 (3.5%)
Injury Severity Score, n (%)	
$\ge$ 0, < 10	344 (38.6%)
$\ge$ 10, < 20	168 (18.9%)
$\ge$ 20, < 30	192 (21.5%)
$\ge$ 30, < 40	108 (12.1%)
$\ge$ 40, < 50	25 (2.8%)
$\ge$ 50, < 60	38 (4.3%)
$\ge$ 60	16 (1.8%)
Trauma type, n (%)	
Penetrating	385 (43.2%)
Blunt	506 (56.8%)
Traumatic brain injury, n (%)	343 (38.5%)

### Statistical model

To uncover the effects of Factor II and D-Dimer on early trauma survival, we employ a joint survival model [11–13]. Joint survival models relate the effects of time-dependent covariates, such as measured clinical biomarkers, on time-to-event data, such as death, accounting for irregular measurement times and intrinsic measurement variability. Recently, joint survival models have been used to study survival in a variety of other diseases [14, 15]. In particular, they have gained prominence due to their ability to robustly model how the continuous evolution of biomarkers affects survival. In the following, we describe the two subcomponents of the joint model: the longitudinal submodels and the survival submodel. We note that for applying this model, we first apply the base-2 log to values of D-Dimer and henceforth refer to this quantity as log D-Dimer.

#### Longitudinal submodels

The longitudinal submodels describe how each time-dependent covariate evolves over the observation window. By explicitly specifying the form, as opposed to naively imputing values, we can account for measurement variability when associating the covariate to the survival outcome. This has been shown to reduce bias in estimates [12] compared to traditional treatments of time-dependent covariates in survival models.

Let $y_{ij}(t)$ denote the measured activity level of coagulopathic biomarker *j* for patient *i* at time *t*. For our study, the longitudinal biomarkers Factor II and D-Dimer are modeled using generalized linear mixed effects models with grouping at the individual level. Specifically, we set $y_{ij}(t) \sim {\mathcal {N}}(\eta _{ij}(t), \sigma _F)$, with

$$\begin{aligned} \eta _{ij}(t) = \beta _{0j} + \beta _{1ij} + \beta _{2j} t + \beta _{3ij} t + \beta _{4l_ij} t + \sum _{k} \beta _{5jk} x_{ik}, \end{aligned}$$

the expected value of the respective marker at time *t* for patient *i*, and $\sigma _F$ the estimated standard deviation. In this formulation, $\beta _{0j}$ and $\beta _{1ij}$ denote the population and individual level intercepts and $\beta _{2j}$ and $\beta _{3ij}$ denote the population level and individual level slopes. $\beta _{4jk}$ specifies the effect of the *k*-th fixed covariate on the *j*-th time-dependent biomarker. The included fixed covariates are age, sex, injury severity score, traumatic brain injury, and injury type, selected due to their relevance in other studies in this area. This is equivalent to fitting a regression line to each of the coagulopathic biomarkers.

#### Survival submodel

The survival submodel connects the longitudinal submodel to the observed patient outcomes. We use the standard proportional hazards model. For each patient, we have a tuple $(T_i, D_i)$ indicating the time that the patient died or was censored and the binary outcome of death. Let $h_i(t)$ be the hazard function for the *i*-th patient at time *t*,

$$\begin{aligned} h_i(t) = h_0(t)\exp \left( \sum _{k} \gamma _{k} x_{ik} + \sum _{j} \alpha _{j} \eta _{ij}(t)\right) , \end{aligned}$$

with $h_0(t)$ the baseline hazard function, $\alpha _j$ the coefficient indicating the strength of the association between longitudinal covariate *j* and survival, and $\gamma _k$ the strength of the association between fixed covariate *k* and survival. The baseline hazard $h_0(t)$ was selected to be a 6-th order B-Spline, as this choice offers maximum flexibility in fitting the unique survival curves of subgroups while avoiding overparameterization [16]. This hazard function at time *t* can be interpreted as the instantaneous rate at which the subject accumulates hazard toward the outcome, assuming that they have survived up to time *t*. Compared to standard time-dependent survival models, the hazard function depends on the expected value of the time dependent covariate, as opposed to the observed or imputed value. The hazard function is linked to the time of the outcome via the survival function

$$\begin{aligned} S(t) = P(T_i \ge t) = \exp \left( -\int _{0}^{t} h_i(x) dx\right) . \end{aligned}$$

For interpretation, we observe the association strengths, $\alpha$, which indicate the change in survival odds for every unit change in the covariate.

#### Estimation

We use the rstanarm package [17, 18] and the joint model function to obtain a Bayesian fit for our model to the data. To estimate the patient-level effects in the longitudinal covariates, we use hierarchical priors to induce shrinkage in the case of few observations [19]. Posterior predictive checks were performed on the longitudinal trajectories to verify that the resulting fit were consistent with the observed data and convergence metrics were checked to validate that the chains were consistent. Analysis was performed using 4000 posterior draws over 4 chains.

## Results

### Factor II and D-Dimer trajectories

**Fig. 2:**
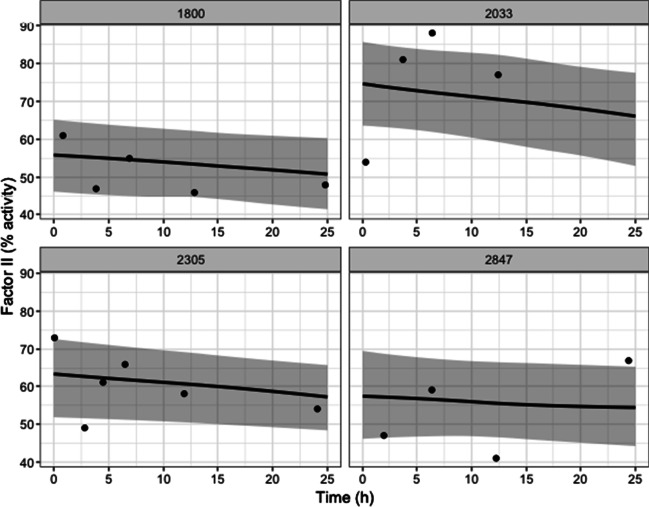
Sample Factor II patient trajectories. Each plot shows a random patient along with their estimated mean trajectory for Factor II and confidence intervals of the mean trajectory. Scattered points are observed data points

**Fig. 3:**
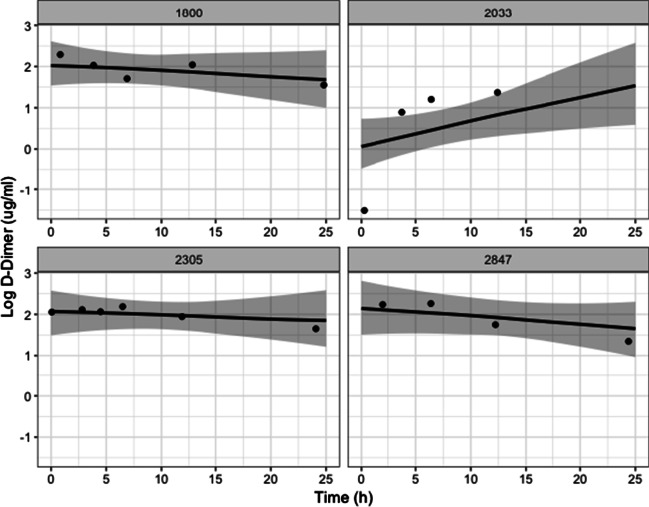
Sample log D-Dimer patient trajectories. Each plot shows a random patient along with their estimated mean trajectory for log D-Dimer and confidence intervals of the mean trajectory. Scattered points are observed data points

In Table 2 we show the estimated fixed effect coefficients for Factor II and log D-Dimer in the longitudinal submodels. Traumatic brain injury is tied to significantly higher levels of both covariates while penetrating injuries tend to decrease the predicted log D-Dimer levels. Higher injury severity score and age slightly increase the level of log D-Dimer and decrease the level of Factor II. These effects indicate that older and more severely injured patients have higher D-Dimer and lower Factor II, which would be intuitive as they indicate higher levels of fibrinolysis and lower levels of available pro-coagulants. Penetrating injuries provide an uncertain effect on Factor II but are associated with lower levels of log D-Dimer. At the population level, Factor II tends to decrease over time while D-Dimer tends to increase. For healthy patients, these would be the expected patterns as clotting factors are used and fibrin degradation products are produced. Figures 2 and 3 show the estimated mean Factor II and log D-Dimer trajectories for 4 patients. Crucially for diagnosis, the 4 patients show varying individual behavior but also reversion to the population level distribution in the case of patients with few measurements.

**Table 2:** Coefficients for longitudinal biomarkers

	Factor II		Log D-Dimer	
	Coefficient	95% credible interval	Coefficient	95% credible interval
Intercept	84.03	80.04, 88.04	$-$ 0.64	$-$ 0.41, $-$ 0.18
Slope	$-$ 0.23	$-$ 0.30, $-$ 0.17	0.011	0.008, 0.016
Age	$-$ 0.15	$-$ 0.21, $-$ 0.10	0.008	0.004, 0.011
Sex (ref: male)	$-$ 1.58	$-$ 4.07, 0.90	$-$ 0.20	$-$ 0.34, $-$ 0.06
Injury severity score	$-$ 0.37	$-$ 0.44, $-$ 0.30	0.043	0.039, 0.047
Traumatic brain injury (ref: yes)	2.65	0.18, 5.07	0.44	0.30, 0.59
Penetrating injury (ref: yes)	$-$ 0.28	$-$ 2.73, 2.10	$-$ 0.25	$-$ 0.38, $-$ 0.11

### Factor II and log D-Dimer associations with survival

Estimated association strengths, interpreted as the increase in odds for every unit increase in the biomarker, as well as 95% credible intervals are shown in Table 3. For exogenous covariates, we find minimal evidence that a higher initial injury severity score and age increases the risks of death. The large uncertainty in the gender hazard ratio is likely due to an insufficient sample size of women in the dataset. As previously known, we find that traumatic brain injury has an extremely large effect on the risk of early death. Interestingly, penetrating injuries seem to significantly increase the risk of early death (hazard ratio [6.08, 3.37–11.19]), however, the large credible intervals indicate a relative lack of data for patients who ultimately died.

**Table 3:** Median and 95% credible interval for hazard ratios

	Hazard ratios	95% credible interval
Factor II	0.94	0.91, 0.96
Log D-Dimer	2.22	1.57, 3.28
Age	1.02	1.01, 1.03
Sex (ref: male)	0.76	0.45, 1.32
Injury severity score	1.03	1.01, 1.04
Traumatic brain injury (ref: yes)	2.71	1.51, 5.04
Penetrating injury (ref: yes)	6.08	3.37, 11.19

For the longitudinal coagulopathic covariates, we find that unit increases in log D-Dimer significantly increase the risk of early death (hazard ratio [2.22, 1.57–3.28]). At the same time, unit increases in Factor II only marginally decrease the risk of death (hazard ratio [0.94, 0.91–0.96]) but with high certainty. This is in good agreement with [20] that concludes that high log D-Dimer levels are the more definitive predictor of death regardless of fibrinogen levels. The significant effect of log D-Dimer suggests that maintaining or lowering the rate of fibrinolysis and thus D-Dimer generation is a key component in reducing the risk of early death in a hospital setting.

### Variation among longitudinal trajectories

**Fig. 4:**
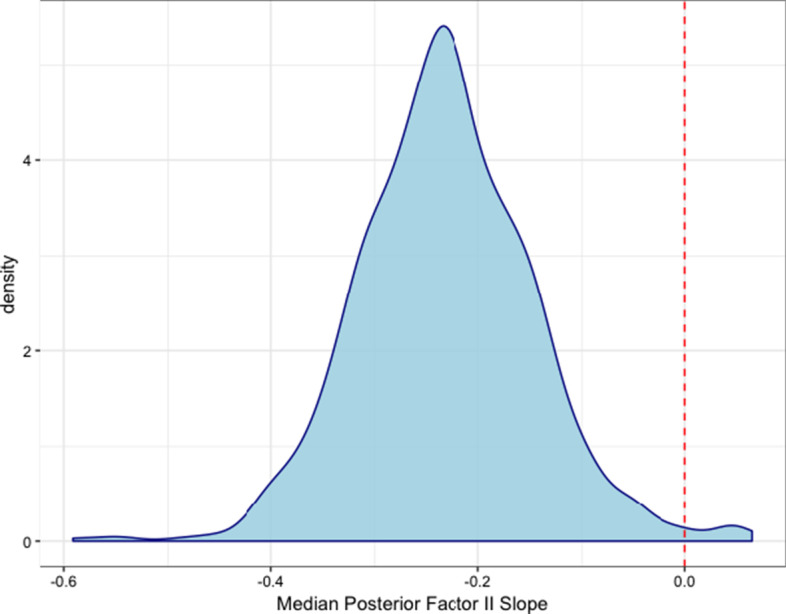
Distribution of median Factor II slopes Estimated median slopes of Factor II for each patient. Red, dashed line indicates the zero-line

**Fig. 5:**
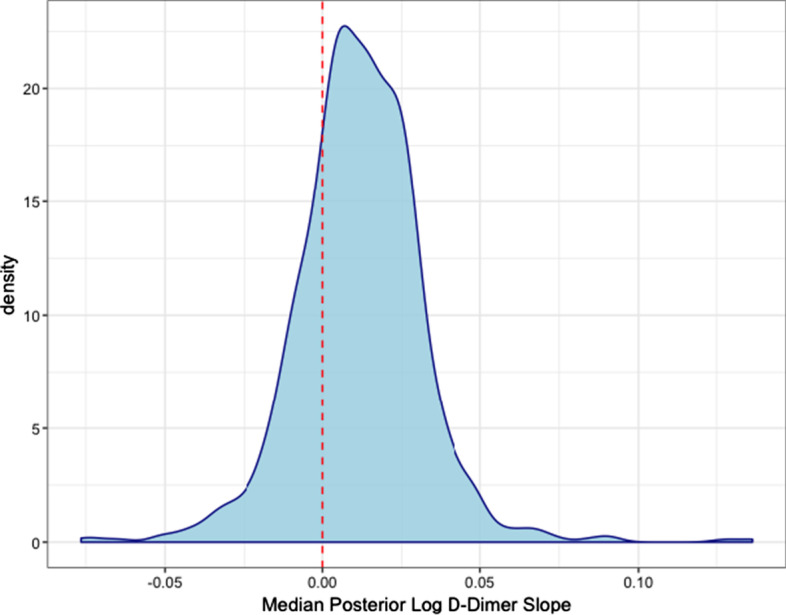
Distribution of median log D-Dimer slopes Estimated median slopes of log D-Dimer for each patient. Red, dashed line indicates the zero-line

In addition to associations, our model estimates individual trajectories for each patient. In this cohort, the vast majority of patients gradually decrease in Factor II levels over the 25 h window, as shown by the distribution of median slopes in Fig. 4. The relatively low rate seems to indicate that, for the majority of patients, Factor II is being held relatively consistent in this 25 h window. We see no cases where the model indicates that Factor II is being consumed at a significantly large rate. In comparison, for log D-Dimer trajectories, we observe large variation from expected behavior. As shown in Fig. 5, patients are centered around 0 but have significant probability mass at both increasing and decreasing D-Dimer levels. However, as D-Dimer is only a product of fibrinolysis, it is difficult to predict what a traditionally healthy trajectory would consist of.

The level of variation among patients, controlling for all of the fixed covariates, indicates that these biomarkers are likely subject to some unaccounted for patient level variability. From a treatment perspective, the varying trends among patients indicate that when making risk assessments for a particular patient, it is important to understand both the estimated hazard ratio as well as the projected trajectory of their biomarkers. As an example, if a patient exhibits high D-Dimer but it is seemingly decreasing, perhaps treatment for fibrinolysis is not necessary.

## Discussion

### Hazard ratios

The hazard ratios indicate that, in this cohort, unit increases in Factor II levels only marginally increase survival odds, while a doubling of D-Dimer (due to the log transformation) largely affects survival odds. Observing the data, a doubling of D-Dimer is not uncommon. Thus, although both consumptive coagulopathy and hyperfibrinolysis do seem to affect survival in some regard, increased rates of fibrinolysis are much more likely to be damaging to survival. From this perspective, greater benefit would be gained by controlling hyperfibrinolysis rather than further managing or increasing factor availability levels.

High levels of D-Dimer have often been associated with poor patient outcomes as a proxy for hyperfibrinolysis. This is further consistent with the growing literature which indicates the importance of addressing hyperfibrinolysis in TIC. Hyperfibrinolysis is estimated to occur in a large number of trauma cases, often with significantly higher mortality rates [21]. The relatively low, but positive impact of Factor II levels suggests that managing Factor II levels is not a significant problem in this cohort. Indeed, the importance of coagulation consumption has long been studied [22] and linked to poor outcomes. As this is a single hospital, retrospective study, it is possible that monitoring and treatment for factor depletion is better monitored and maintained, leading to better outcomes for patients that exhibit signs of coagulopathy.

Also of note is the significant effect of both traumatic brain injury and penetrating trauma, independent of the levels of both Factor II and D-Dimer. Largely, the increased mortality from injuries of this types are well known in trauma [23]. The scale of the hazard ratios provides a rough perspective on the priority of treatment, with concern based on the the type of injury preceding further monitoring of hyperfibrinolysis and finally consumptive coagulopathy.

### D-Dimer modulation in trauma care

D-Dimer, while often used as a surrogate for measuring fibrinolysis, can also be affected by other factors. Due to the risk associated with high levels of D-Dimer as indicated by our model, it is important to further describe some of these alternative factors.

From a physiological perspective, as D-Dimer is a protein fragment created from the breakdown of a fibrin clot, any processes which affects the rate of clot breakdown could result in measured changes in D-Dimer levels. In ICU patients, while initially elevated levels of D-Dimer are expected due to the nature of the injuries involved, the type of injury can have a significant effect on D-Dimer levels over time the condition of the patient evolves. As seen in Table 3, patients with non-penetrating or traumatic brain injuries tend to see an increase in D-Dimer levels over time. Typically, for healthy patients recovering from injury, coagulation and fibrinolysis are expected to slow down, resulting in declining D-Dimer levels. Sathe et al. [24] further mentions several non-hyperfibrinolytic pathological and non-pathological conditions which have also been shown to increase D-Dimer. An important possibility that may affect D-Dimer levels without clearly indicating increased fibrinolysis is the decreased ability to clear D-Dimer from the blood, as has been found in patients with liver disease and cirrhosis [25]. In these cases, an underlying liver problem may result in abnormally high levels of D-Dimer as it accumulates over time, even when the patient is not hyperfibrinolytic.

Common interventions may also cause D-Dimer levels to change. A recent standard treatment for hyperfibrinolysis is administration of the anti-fibrinolytic drug, Tranexamic acid (TXA). As it’s mechanism of action is to prevent plasmin formation and thus slow down fibrinolysis, it naturally decreases D-Dimer levels. This has been demonstrated in both laboratory and clinical settings [26, 27] with time-scales as short as 30 minutes after administration [28].

### Clinical considerations

Our findings broadly suggest that, from an early clinical perspective, managing fibrinolysis is typically more of a concern than managing consumptive coagulopathy over a 24 h window of care. Furthermore, as shown in Figs.4 and 5, the trends of these factors can vary significantly between different patients and thus treatment and evaluation of patient state can possibly improve by projecting how a patients’ state is trending. This follows exactly the thinking of the clinician where they are constantly evaluating current physiologic/biologic state of a patient and trying to predict and modify trajectory. Although high D-Dimer levels are linked to poor outcomes, if the patient is projected to be improving, further treatment may not be necessary. The development of explicit risk metrics which provide individual projected trajectories as such could provide valuable information in acute decision making.

As the factors analyzed in this work are not typically measured in real-time, our work primarily aims to explore the risk factors in TIC and to observe patient-level variations over their ICU stay. State of the art treatment of TIC in the ICU typically includes providing blood products such as crystalloids, fresh frozen plasma, and packed red blood cells through transfusion and by administering drugs such as Tranexamic acid [26, 29] both of which aim to control hyperfibrinolysis as well as consumptive coagulopathy. A significant amount of recent research has focused on implementing better protocols for these interventions using viscoelastic assays, such as TEG and ROTEM [30, 31]. These measurements aim to provide a more holistic picture of blood coagulation, which can lead to significant advantages in accuracy or diagnosis of coagulation malfunctions. Additionally, in the future, we expect that results can be extracted at the point-of-care and used for a truly precision medicine individualized approach to diagnosis and treatment.

The use of viscoelastic measurements in a similar computational study can extend the conclusions of this work to more precisely capture malfunctions of the coagulation system as well as provide for a practical component in a dynamic risk-prediction system that can aid in acute decision making over a patient’s stay.

### Model limitations

Importantly, there are a few limitations to full interpretation of this model. Due to the retrospective and single hospital nature of the data, these results can be understood more as an evaluation of early trauma hospital protocol. As interventions such as mass transfusion are not accounted for, from this perspective, we find that the trauma protocol mediates the effects of most covariates but does not seem to adequately control for the effects of increasing log D-Dimer levels. To improve interpretation, we would need to utilize data from multiple hospitals. Furthermore, certain studies indicate that elevated log D-Dimer is not necessarily a definitive sign of hyperfibrinolysis [32] and can be rather thought of as a confounded measure of injury severity and the need for an activated coagulation system. Thus, utilization of viscoelastic assays, such as TEG and ROTEM, that offer different measurements may help to better distinguish the effect of the two components of coagulation on survival. Despite this however, our data show that D-dimer, whatever its biologic meaning (fibrinolysis or enhanced clot breakdown) is an important marker of future mortality. Similarly, use of other proteins in the coagulation cascade may reveal more informative results with respect to how much of an impact consumptive coagulopathy over time actually has on survival odds. A secondary model for interventions may also help for improving treatment for TIC.

## Conclusions

We fit a joint-survival model to trauma to quantify the effect of activity levels of Factor II and log D-Dimer on survival in a early 25 h window. From this work, we find that increases in Factor II levels have a small, but positive effect on survival, while increases in log D-Dimer levels have a large negative effect on survival. The nature of this study suggests further investigation into methods to prevent excessive fibrinolysis in hospital protocol. Furthermore, this model can also be used to better understand individualized and dynamic risk prediction from a standard patient, due to the large variability in patient longitudinal trajectories.

## Data Availability

The datasets generated and/or analysed during the current study are not publicly available due to their containing information that could compromise the privacy of research participants but are available from the corresponding author on reasonable request.

## Abbreviations

TIC: Trauma-induced coagulopathy
ICU: Intensive care unit
TEG: Thromboelastography
ROTEM: Rotational thromboelastometry

## References

[1] Cohen MJ, Christie SA (2017). Coagulopathy of trauma. Crit Care Clin.

[2] Cohen MJ, Kutcher M, Redick B, Nelson M, Call M, Knudson MM, Schreiber MA, Bulger EM, Muskat P, Alarcon LH (2013). Clinical and mechanistic drivers of acute traumatic coagulopathy. J Trauma Acute Care Surg.

[3] Spahn DR, Bouillon B, Cerny V, Coats TJ, Duranteau J, Fernández-Mondéjar E, Filipescu D, Hunt BJ, Komadina R, Nardi G (2013). Management of bleeding and coagulopathy following major trauma: an updated European guideline. Crit Care.

[4] Brummel-Ziedins KE, Orfeo T, Callas PW, Gissel M, Mann KG, Bovill EG (2012). The prothrombotic phenotypes in familial protein c deficiency are differentiated by computational modeling of thrombin generation. PLoS ONE.

[5] Loof T, Deicke C, Medina E (2014). The role of coagulation/fibrinolysis during streptococcus pyogenes infection. Front Cell Infect Microbiol.

[6] Dobson GP, Letson HL, Sharma R, Sheppard FR, Cap AP (2015). Mechanisms of early trauma-induced coagulopathy: The clot thickens or not?. J Trauma Acute Care Surg.

[7] Kutcher ME, Ferguson AR, Cohen MJ (2013). A principal component analysis of coagulation after trauma. J Trauma Acute Care Surg.

[8] Zhang Y, Wu TB, Daigle BJ, Cohen M, Petzold L (2016). Identification of disease states associated with coagulopathy in trauma. BMC Med Inform Decis Mak.

[9] Weitz JI, Fredenburgh JC, Eikelboom JW (2017). A test in context: D-dimer. J Am Coll Cardiol.

[10] Holcomb JB, Del Junco DJ, Fox EE, Wade CE, Cohen MJ, Schreiber MA, Alarcon LH, Bai Y, Brasel KJ, Bulger EM (2013). The prospective, observational, multicenter, major trauma transfusion (PROMMTT) study: comparative effectiveness of a time-varying treatment with competing risks. JAMA Surg.

[11] Rizopoulos D, Ghosh P (2011). A Bayesian semiparametric multivariate joint model for multiple longitudinal outcomes and a time-to-event. Stat Med.

[12] Tsiatis AA, Davidian M. Joint modeling of longitudinal and time-to-event data: an overview. Stat Sin. 2004;809–834.

[13] Ibrahim JG, Chu H, Chen LM (2010). Basic concepts and methods for joint models of longitudinal and survival data. J Clin Oncol.

[14] Proust-Lima C, Dartigues J-F, Jacqmin-Gadda H (2016). Joint modeling of repeated multivariate cognitive measures and competing risks of dementia and death: a latent process and latent class approach. Stat Med.

[15] Sweeting MJ, Barrett JK, Thompson SG, Wood AM (2017). The use of repeated blood pressure measures for cardiovascular risk prediction: a comparison of statistical models in the aric study. Stat Med.

[16] Brilleman SL, Crowther MJ, Moreno-Betancur M, Buros Novik J, Dunyak J, Al-Huniti N, Fox R, Hammerbacher J, Wolfe R. Joint longitudinal and time-to-event models for multilevel hierarchical data. Stat Methods Med Res. 2018;0962280218808821. 10.1177/0962280218808821 30378472

[17] Stan Development Team: Rstanarm: Bayesian applied regression modeling via stan. r package version 2.17.4. 2018.

[18] Carpenter B, Gelman A, Hoffman MD, Lee D, Goodrich B, Betancourt M, Brubaker M, Guo J, Li P, Riddell A (2017). Stan: a probabilistic programming language. J Stat Softw.

[19] Gelman A, Carlin JB, Stern HS, Dunson DB, Vehtari A, Rubin DB (2013). Bayesian data analysis.

[20] Hayakawa M, Maekawa K, Kushimoto S, Kato H, Sasaki J, Ogura H, Matauoka T, Uejima T, Morimura N, Ishikura H (2016). High d-dimer levels predict a poor outcome in patients with severe trauma, even with high fibrinogen levels on arrival: a multicenter retrospective study. Shock.

[21] Schöchl H, Voelckel W, Maegele M, Solomon C (2012). Trauma-associated hyperfibrinolysis. Hämostaseologie.

[22] Kashuk JL, Moore EE, Millikan JS, Moore JB (1982). Major abdominal vascular trauma—a unified approach. J Trauma.

[23] Tong W-S, Zheng P, Zeng J-S, Guo Y-J, Yang W-J, Li G-Y, He B, Yu H, Li Y-S, Tang X-F (2012). Prognosis analysis and risk factors related to progressive intracranial haemorrhage in patients with acute traumatic brain injury. Brain Inj.

[24] Sathe PM, Patwa UD (2014). D Dimer in acute care. Int J Critl Illness Inj Sci.

[25] Gram J, Duscha H, Zurborn KH, Bruhn HD (1991). Increased levels of fibrinolysis reaction products (D-dimer) in patients with decompensated alcoholic liver cirrhosis. Scand J Gastroenterol.

[26] Roberts I, Shakur H, Coats T, Hunt B, Balogun E, Barnetson L, Cook L, Kawahara T, Perel P, Prieto-Merino D, Ramos M, Cairns J, Guerriero C (2013). The CRASH-2 trial: a randomised controlled trial and economic evaluation of the effects of tranexamic acid on death, vascular occlusive events and transfusion requirement in bleeding trauma patients. Health Technol Assess.

[27] Picetti R, Shakur-Still H, Medcalf RL, Standing JF, Roberts I (2019). What concentration of tranexamic acid is needed to inhibit fibrinolysis? A systematic review of pharmacodynamics studies. Blood Coagul Fibrinol.

[28] Shakur-Still H, Roberts I, Fawole B, Kuti M, Olayemi OO, Bello A, Huque S, Ogunbode O, Kotila T, Aimakhu C, Okunade OA, Olutogun T, Adetayo CO, Dallaku K, Mansmann U, Hunt BJ, Pepple T, Balogun E (2018). Effect of tranexamic acid on coagulation and fibrinolysis in women with postpartum haemorrhage (WOMAN-ETAC): a single-centre, randomised, double-blind, placebo-controlled trial. Welcome Open Res.

[29] Ishikura H, Kitamura T (2017). Trauma-induced coagulopathy and critical bleeding: the role of plasma and platelet transfusion. J Intensive Care.

[30] Gonzalez E, Moore EE, Moore HB, Chapman MP, Chin TL, Ghasabyan A, Wohlauer MV, Barnett CC, Bensard DD, Biffl WL (2016). Goal-directed hemostatic resuscitation of trauma-induced coagulopathy: a pragmatic randomized clinical trial comparing a viscoelastic assay to conventional coagulation assays. Ann Surg.

[31] Gonzalez E, Moore EE, Moore HB (2017). Management of trauma-induced coagulopathy with thrombelastography. Crit Care Clin.

[32] Raza I, Davenport R, Rourke C, Platton S, Manson J, Spoors C, Khan S, De’Ath H, Allard S, Hart D (2013). The incidence and magnitude of fibrinolytic activation in trauma patients. J Thromb Haemost.

